# How an Insurance Agent Was Beaten

**Published:** 1874-12

**Authors:** 


How an Insurance Agent was Beaten.—Between Kenosha and Milwaukee, says a Wisconsin paper, an agent of a Traveler's Insurance Company entered a car, and having issued tickets to several of the passengers, approached an elderly lady, who it afterwards appeared was deaf.

"Madame, would you like to insure against accident?" inquired the agent.

"I'm going to Oshkosh, to visit my darter, who is married up there, and has got a baby."

The agent raised his voice a little.

"Would you like to insure your life against accident?"

"She's married two years and a half. It's a gal."

Agent still louder.

"I'm an insurance agent, madam. Don't you want your life insured against accident?"

"Oh! I didn't understand you," said the old lady. "No; her name is Johnson; my name is Evans, and I live five miles from Kenosha."

The agent vanished.

